# Coherent perfect loss with single and broadband resonators at photonic crystal nanobeam

**DOI:** 10.1515/nanoph-2023-0788

**Published:** 2024-01-25

**Authors:** Jihoon Choi, Young Ki Hong, Heeso Noh

**Affiliations:** Department of Physics, Kookmin University, Seoul 02707, Republic of Korea; Research Institute of Natural Science, Gyeongsang National University, Jinju 52828, Republic of Korea; Department of Physics, Gyeongsang National University, Jinju 52828, Republic of Korea

**Keywords:** coherent perfect absorption, photonic crystal, broadband resonator, nanobeam

## Abstract

Coherent perfect absorption (CPA) has been studied in various fields, such as metasurface, photonics, and acoustics, because of its ability to perfectly absorb light at a specific wavelength. However, the narrow bandwidth of CPA makes its application to on-chip photonics challenging. This limitation can be overcome by using a broadband resonator. Here, we demonstrate the coherent perfect loss (CPL) with respect to a single and broadband resonator at photonic crystal nanobeam. By using the finite element method, both cases of the CPL were simulated and optimized for the single and broadband resonators. In the optimized structure, a CPL occurs for both resonators. These results confirm that the perfect loss region for the broadband resonator is wider than that for the single resonator. These results are experimentally verified by fabricating both cases of CPL cases on a silicon-on-insulator by using electron beam lithography. An almost perfect loss of more than 95 % is observed for both single and broadband CPLs. Furthermore, the almost perfect loss region at the broadband resonator broadens more than that at the single resonator. The optimized structure for CPL has the potential for easy applications to on-chip photonics, such as optical switches, modulators, sensors, and logic gates.

## 1 Introduction

Coherent perfect absorption (CPA) is time-reversed lasing and can be achieved in resonators if the optical gain for lasing is replaced with an optical loss of the same magnitude. The incident lights are completely absorbed in the resonator [1]. Chong et al. [2] was the first to discover CPA, which was later experimentally demonstrated by Wan et al. [3]. Since then, CPA has attracted considerable research attention in various fields, such as on-chip photonics [4], metasurfaces [5], [6], [7], photonic crystals slab [8], and parity-time symmetric systems [9]. In addition, CPA has been extensively reported in various platforms and applications, such as disordered media [10], single-port interferometers [11], [12], optical switches [13], sensors [14], [15], logic gates [16], and arbitrary wavefronts [17].

At typical CPA, light is perfectly absorbed by a resonator with a high quality factor (Q-factor). It makes the CPA structure sensitive to environmental changes such as temperature. Although the narrow bandwidth of CPA can be utilized for high-sensitivity sensors, it can be tricky for on-chip optical circuits. To overcome this limitation, CPA with a broadband resonator could be adopted. The broadband CPA has been studied from various aspects, including plasmonic effects [18], [19], [20], metallic nanoparticles [21], thin-film [22], epsilon-near-zero materials [23], [24], [25], parity-time symmetric structures with exceptional point [26], and metasurface [27], [28], [29], [30]. By using analytical calculations and simulations, studies have demonstrated broadband CPA for a multi-layer structure [31], [32]. In previous studies, broadband CPA was achieved using a resonator with a low Q-factor or thin film such as metasurface, conductive, and multi-layer. Another study presented broadband CPA using the parity-time structure. The thin film is inappropriate to apply for on-chip photonics, and the simple fabrication procedure is essential for on-chip photonics.

For the photonic crystal nanobeam (PCN), the multi-layer structure can be achieved by changing the hole size because the effective refractive index of PCN can be adjusted by modifying the hole size at PCN [33], [34]. PCN has been extensively studied because its fabrication process is relatively simple and it can achieve high performance. The PCN has been studied in various applications, such as in filters [35], modulators [36], circulators [37], switches [38], sensors [39], [40], [41], phase shifters [42], and resonators [43], [44], [45], [46], [47], [48], [49], [50], [51], [52]. The PCN can function as a perfect reflector and functional layer in couplers, scatterers, and layers with different effective refractive indices. In addition, by using a PCN resonator, a high Q-factor can be achieved up to a magnitude of 10^8^ theoretically and of 10^6^ experimentally [53]. Therefore, the PCN resonator can be used in lasing [54], [55], [56] and in CPA. The perfect loss based on CPA at the PCN resonator with a single port has been demonstrated through simulations, in which the PCN served as a coupler and functional reflector [57].

In this study, we demonstrated on-chip coherent perfect loss (CPL) with the broadband PCN resonator. Although CPL is the same phenomenon as CPA, it mainly uses scattering and coupling loss instead of optical absorption. The broadband CPL is based on the multi-layer structure using the photonic crystal nanobeam for on-chip photonics. This structure is simple to fabricate and applicable to on-chip photonics because it uses photonic crystal nanobeam. In the 3-dimensional finite element method (FEM), the structure for CPL is optimized and simulated in the broadband resonator. The perfect loss was determined with the full width at half max (FWHM) of 8.4 nm, and the perfect loss region ranged from 1549 to 1551 nm. The spectral width of the perfect loss at broadband CPL was conformed to be broader than that of CPL with a single resonator. For experimental verification, we fabricated the structure with silicon-on-insulator (SOI) by using electron beam lithography (EBL). In the experiments, an almost perfect loss was achieved above 95 % between 1577 and 1587 nm with an FWHM of approximately 18.3 nm. The proposed broadband CPL can be easily applied to the on-chip integration of optical circuits.

## 2 Simulation

**Figure 1:**
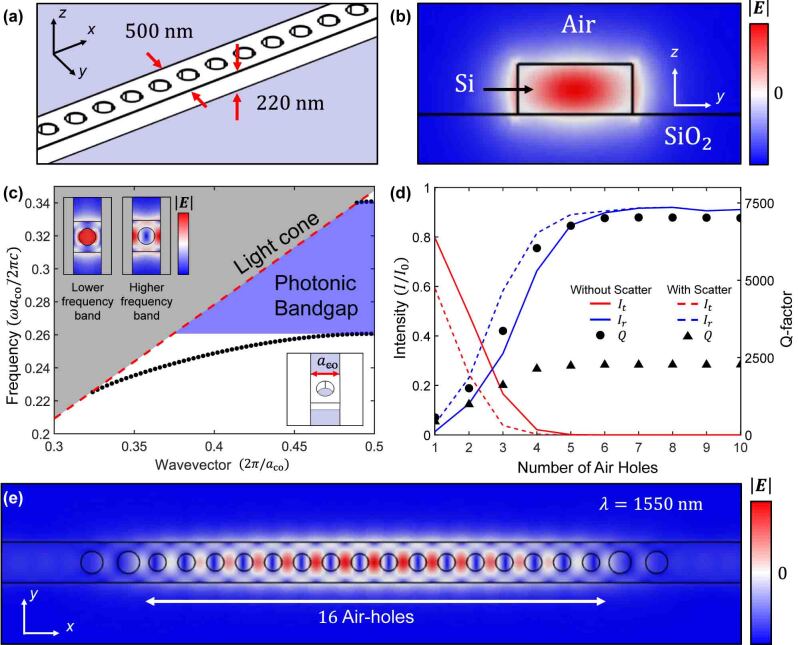
Schematic of the optical nanobeam and PCN comprising in SOI. (a) The optical nanobeam is 220 nm thick and 500 nm wide. In these conditions, the optical nanobeams support only a single mode propagation. (b) Electric field (|*E*|) distribution in optical nanobeam. (c) Dispersion relations of PCN (*TE* bands) with *a*_co_ = 450 nm and *r*_co_/*a*_co_ = 0.3. The insets show the electric field (|*E*|) distribution of each band edge mode at PCN. (d) Transmission intensity (red solid and dashed lines) *I*_*t*_, reflection (blue solid and dashed lines) *I*_*r*_, and the Q-factor (black circle and triangle) of PCN resonator as a function of the number of air holes in a coupler when PCN resonator is without and with a scatterer. (e) Electric field (|*E*|) distribution in a PCN resonator when the resonance occurred at the wavelength of 1550 nm (*λ*_ref_).

In the 3-dimensional simulation, we used the FEM with COMSOL Multiphysics 6.1 [58]. We designed the PCN on SOI, comprising 220-nm-thick Si on an SiO_2_ substrate. The commonly used wavelength in the field of telecommunications, 1550 nm, was used as the reference wavelength, *λ*_ref_. At this wavelength, the refractive indices of the Si and SiO_2_ were 3.48 [59] and 1.44 [60], respectively. In our simulation, PCN supports in-plane electric field mode (*E*_*x*_ and *E*_*y*_). The PCN comprises a waveguide with a width of 500 nm and an air-hole array (Figure 1(a)). Figure 1(b) shows the electric field distribution (|*E*|) in waveguides that support only fundamental modes.

We first simulated a single PCN resonator, comprising 20 air holes, 16 of which located in the center structure serve as the resonator. In addition, two air holes on each side of the resonator serve as the coupler to control the light leakage from the resonator. The geometry of the air-hole array is defined as lattice constant, *a*, and the ratio of hole radius, *r*, to *a*, *r*/*a*. We set lattice constant and the corresponding ratio with respect to the hole radius of the coupler to *a*_co_ = 450 nm and *r*_co_/*a*_co_ = 0.3, respectively, because *λ*_ref_ is in the photonic bandgap in these conditions. Figure 1(c) shows the dispersion relation of the PCN coupler. The width of the photonic bandgap is 405.4 nm, ranging from 1320.8 to 1726.2 nm.

Figure 1(d) shows the Q-factor (black circle), transmission, *I*_*t*_ (red solid line), and reflection, *I*_*r*_ (blue solid line) of the PCN resonator as functions of the number of air holes in the coupler. With the increase in the number of air holes in the coupler, the Q-factor of the resonator increases and the coupling coefficient decreases. Therefore, as the number of air holes increases, *I*_*r*_ increases while *I*_*t*_ decreases. Here, we must determine the appropriate number of air holes in the coupler. For more number of air holes, the coupling strength with respect to the resonators is low. In this situation, *I*_*r*_ is more significant than *I*_*t*_ when the number of air holes is more than three. Therefore, the condition of CPA cannot be satisfied in the single resonator with a coupler of more than three air holes. In addition, resonators with a coupler and a few number of air holes result in a high coupling strength and low-Q factors; consequently, high absorption is required to satisfy the CPA condition. In our structure, the optimized condition includes two air holes at each side of the resonator.

Herein, we determined the resonance by modifying the geometry of the air holes. The resonance wavelength can be controlled by modifying the lattice constant of the air-hole array (*a*_res_) and radius of the air holes of the PCN resonator (*r*_res_) because the whole length and effective refractive index of the PCN resonator change with the geometry of the air-hole array. When *a*_res_ = 354.5 nm and *r*_res_/*a*_res_ = 0.3, the resonance occurred at *λ*_ref_. Figure 1(e) shows the electric field distribution in the PCN resonator. Because the resonance displays an even symmetry, the two incident lights should be in-phase for CPA.

To satisfy the conditions of CPA, the resonator must have the same amount of absorption as a gain of the lasing threshold. Here, we should match the loss condition instead of absorption because both Si and SiO_2_ have no optical absorption at *λ*_ref_. We locate the two waveguides as scatterers at the center of the resonator in the *y* direction to achieve the conditions of CPL. In this configuration, the total loss of the resonator is the sum of the scattering loss at the scatterer and coupling loss at the coupler. The width of the scatterer is 1500 nm, which is sufficient for scattering the light from the resonator. If we adjust the distance between the scatterer and resonator, *d*_s_, we can control the total loss of the structure.

The resonance wavelength changes with *d*_s_ because the scatterer perturbs the effective refractive index of the PCN resonator. This phenomenon is similar to the photonic crystal slab coupled with microfiber [61]. Therefore, we must modify the *a*_res_ to match the resonance wavelength to *λ*_ref_. When the PCN resonator is with the scatterer (*d*_s_ = 140 nm), the Q-factor of the PCN resonator decreases, as depicted in Figure 1(d) (black triangles). The *I*_*t*_ (red dashed line) and *I*_*r*_ (blue dashed line) at the PCN resonator with respect to the scatterer are shown in Figure 1(d). When the scatterer is located around the PCN resonator, the coupling strength decreased, thus resulting in the decrease of *I*_*t*_ and increase of *I*_*r*_.

**Figure 2:**
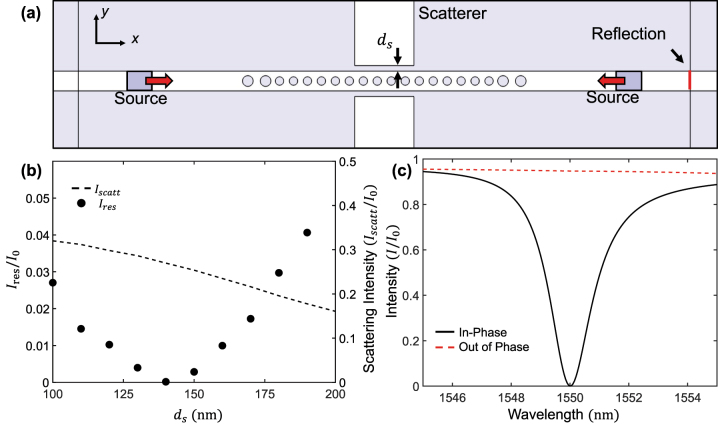
Simulation for CPL with PCN resonator. (a) Simulation configuration for CPL. The structure comprises a PCN resonator, coupler, and scatterer. Two lights with the same amplitude and phase are incident from a unidirectional source from each side of the nanobeam. (b) The normalized intensity of reflection at resonance (*I*_*res*_/*I*_0_, black circles) and the normalized scattering intensity from scatterer ( $I_{scatt}/I_{0}$ , dashed line) as a function of *d*_s_. At the condition of CPL, the reflection intensity reaches zero. (c) The normalized intensity of reflection from the resonator with *d*_s_ = 140 nm as a function of wavelength when the two incident lights are in-phase (black solid line) and out of phase (red dashed line), respectively.

Figure 2(a) shows the simulation configuration for CPL. Two identical light sources are located on both sides of the waveguide at the same distance from the resonator. Thus, the two incident lights are in-phase. The incident light demonstrates only one-way directional propagation using the unidirectional incident wave [62]. Here, two incident lights propagate toward the resonator along the *x*-axis. The red line in Figure 2(a) represents the detector that records the intensity of the reflected light. As this structure has the mirror-symmetry at the center of the resonator, the reflection intensity at both side ends is equal.

The normalized intensity of the reflected light at resonance wavelength, *I*_res_/*I*_0_, as a function of *d*_s_ is depicted in Figure 2(b) (black circles), where *a*_res_ changes with respect to *d*_s_. As *d*_s_ increases, scattering intensity from the scatterer is decreased (dashed line in Figure 2(b)); this means that the loss of resonator can be modified by the distance between the resonator and scatterer. The scattering loss decreases with an increase in *d*_s_ from 100 nm. If the scatterer is very close to the PCN resonator, the loss condition of CPL is mismatched. Thus, when the scatterer is located at the appropriate distance from the resonator, *I*_res_/*I*_0_ approaches 0. This implies that the loss condition of CPL is satisfied. The loss condition is satisfied when *d*_s_ = 140 nm. Figure 1(d) confirms that *I*_*t*_ and *I*_*r*_ are the same when *d*_s_ = 140 nm, and the number of air holes at the coupler is two. Under this condition, *a*_res_ = 353.8 nm for resonance at *λ*_ref_. *I*_res_/*I*_0_ increases as *d*_s_ moves away from the CPL condition.

Figure 2(c) shows the intensity of the reflected light from the resonator with *d*_s_ = 140 nm as a function of the wavelength when the two incident lights are in-phase (black solid line) and out of phase (red dashed line), respectively. When the CPL conditions are satisfied, we can confirm the perfect loss at *λ*_ref_ with the FWHM of 1.76 nm. There is almost no loss when the two incident lights are out of phase. Because our system is based on CPA, the optical loss in structure is sensitive to the relative phase between two incident lights.

**Figure 3:**
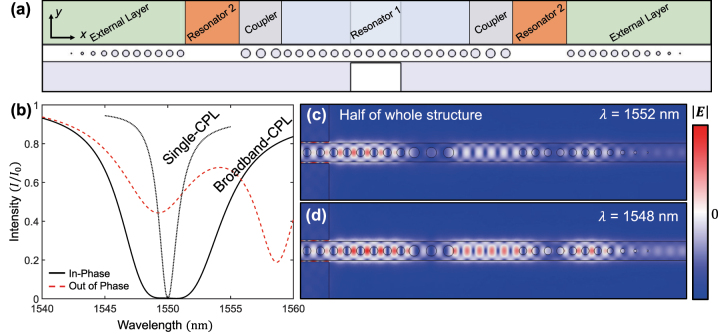
Simulation for broadband CPL using multi-layered PCN resonator. (a) Configuration for broadband CPL. The structure comprises multiple layers, two resonators, couplers, and external layers, which consists of PCN with various sizes of holes. (b) Intensity of reflection for CPL with broadband (black solid line) and single resonators (black dotted line) when the two incident lights are in-phase. The perfect absorption region and FWHM of the broadband CPL are broadened than those of single CPL. The red dashed line depicts the intensity of reflection when the two incident lights are out of phase. (c) and (d) Electric field (|*E*|) distribution for resonance of perfect absorption at broadband CPL. The electric field distribution at two wavelengths shows similar profiles.

The broadband CPL structure can be formed using multilayered PCN with various-sized air holes. Figure 3(a) shows the multilayered PCN resonator. Each layer has a different effective refractive index controlled by hole sizes of PCN. As in the previous simulation, resonator 1 comprises 16 air holes. In broadband resonator cases, the optimized number of air holes for the coupler is three. Further, the resonator 2 is formed by the defect of air holes, wherein the length of resonator 2 is 1493.9 nm. To satisfy the conditions of the broadband resonator, the size of the air holes of the outermost layer (11 air holes at each side), named the external layer, must be modified. The condition of the broadband resonator is that the effective refractive index of each layer, *n*_1_ (resonator 1), *n*_2_ (resonator 2), *n*_ex_ (external layer), is *n*_1_ > *n*_ex_ > *n*_2_ or *n*_1_ < *n*_ex_ < *n*_2_ [31], [32]. According to the effective medium approximations [33], [34], *n*_2_ > *n*_1_. Therefore, *n*_ex_ should satisfy *n*_1_ > *n*_ex_ > *n*_2_, for which the size of the air hole of the external layer should be smaller than that of resonator 1. Here, *a*_ex_ = 326 nm (lattice constant of external layer). At the edge of the external layer, the size of the air holes is tapered because we must reduce the reflection at the boundary.

The simulation for broadband CPL was performed in the same manner as that of the single resonator. To satisfy the conditions of CPL of the broadband resonator, we also adopted the scatterer with a width of 1500 nm. When *d*_s_ = 33.5 nm, the condition of CPL is satisfied. In this situation, *a*_res_ = 350 nm. The intensity of the reflected light as a function of wavelength is depicted in Figure 3(b). The wide, perfect loss region was confirmed to range from 1549 to 1551 nm, with FWHM of loss as 8.4 nm. Compared to CPL with a single resonator (dotted line in Figure 3(b)), the perfect absorption region for the broadband CPL was certainly broadened. Also, when the incident lights are out of phase, the CPL does not arise (red dashed line in Figure 3(b)). The electric field distribution of the resonance modes (at *λ* = 1552 (a) and 1548 nm (b)) at the broadband resonator is depicted in Figure 3(c). The electric field distributions are almost similar at the two resonances despite different wavelengths.

## 3 Experiments

**Figure 4:**
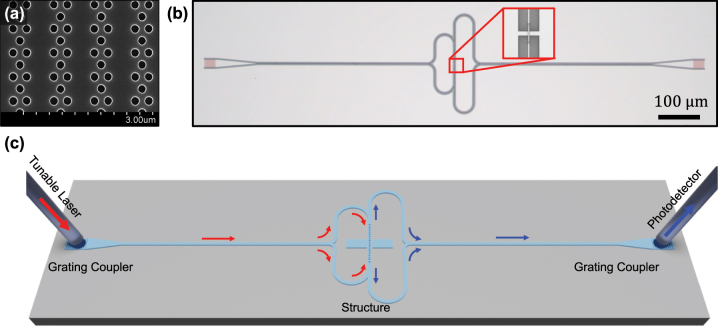
Experimental schematics of CPL in SOI. (a) SEM image of fully etched photonic crystal grating coupler. The grating coupler is optimized using an FEM simulation. (b) Microscopic image for the fabricated structure. Inset shows the magnified image of CPL, which comprises a resonator, coupler, and scatterer. (c) Schematics of the experiment. The light from tunable laser is coupled to the structure by using a grating coupler. Along the waveguide, light is transmitted to the structure. The reflected light is propagated to the output grating coupler and recorded at the photodetector.

For the experimental demonstration, we fabricated the structure for CPL in SOI (Si with 220-nm thickness on SiO_2_) through the Si fabrication procedure by using EBL (JEOL, JBX-9300FS). We adopted the fully etched photonic crystal grating coupler to inject light into the structure [63]. Figure 4(a) shows the scanning electron microscope (SEM, Hitachi, S-4800) image of the photonic crystal grating coupler, displaying a high coupling efficiency and simple fabrication. Figure 4(b) shows the microscopic image (Nikon, LV100ND) of the fabricated whole structure detected by the CMOS sensor (KOPTIC, HK6.3E3S) using a *×*10 objective lens (Nikon, LE Plan 10×). Because the designed CPL structure possesses an even symmetry at the center of the resonator, two incident lights are required with the same phase and intensity. We designed the Y-shaped waveguide that separates symmetrically to satisfy these requirements. Two separated waveguides are connected to each side of the structure with the same path length. The inset of Figure 4(b) shows the magnified structure by using a *×*100 objective lens (Nikon, TU Plan Fluor 100×). To guide the reflected light toward the detector, the waveguides have branches at each side of the resonator. At the branch of the waveguide, the back reflection to the detector is less than 0.1 % of transmission, calculated by FEM simulation. The reflected lights propagate to the detector through these branches. Two waveguides toward the detector were merged into one waveguide connected to the grating coupler.

Figure 4(c) depicts experimental schematics. The incident and outgoing lights are depicted as red and blue arrows, respectively. We used the wavelength tunable laser (Thorlabs, TLK-L1550R) around 1550 nm as the light source. The incident light was coupled with the single-mode fiber (Thorlabs, P3-1550A-FC-5). One end of the fiber was stripped to be directly coupled with a grating coupler. The reflected lights are coupled to the optical fiber from the output grating coupler and recorded at the InGaAs photodetector (Zolix, DInGaAs1700-TE).

**Figure 5:**
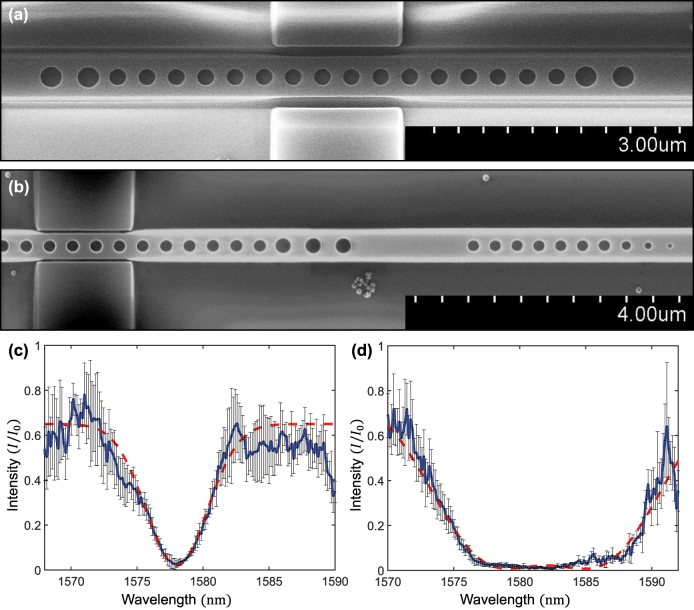
Experimental results of CPL. SEM images of the fabricated CPL with (a) single and (b) broadband resonators. The single CPL comprises a PCN resonator, couplers, and scatterers. The SEM image of the broadband CPL shows resonator 2 and an external layer. The average reflection intensity over the total number of measurements as a function of wavelength at (c) single and (d) broadband CPL (blue solid line). The single CPL shows a perfect loss at 1578 nm of wavelength with 5.4 nm of FWHM. The perfect loss region is broadened to 18.1 nm in FWHM at broadband CPL. The red dashed lines depict the fitting of (c) single Gaussian function and (d) two Gaussian functions, respectively. The error bars show the calculated standard deviation at each wavelength.

From the SEM images in Figure 5(a) and (b), we can confirm that both structures with single and broadband resonators were fabricated well onto the SOI. The blue solid line in Figure 5(c) and (d) depicts the average intensity of the reflection over the total number of recorded at the photodetector. For the single resonator (Figure 5(c)), we observed an almost perfect loss above 95 % at the wavelength of 1578 nm, and the FWHM of loss was 5.4 nm. In contrast, in the broad resonator (Figure 5(d)), the loss above 95 % ranged from 1577 to 1587 nm. Here, the FWHM of loss spectrum range was approximately 18.3 nm. The red dashed lines in Figure 5(c) and (d) show the fitted lines with a Gaussian function. In the broadband CPL, the fitting demonstrates the sum of two Gaussian functions. These results show that the CPL can occur in a PCN with single and broadband resonators, and the multilayered PCN resonator can broaden this perfect loss region. The shift of approximately 20 nm between the wavelength of the simulation and experimental results is attributed to a fabrication error. The widened FWHM of the single resonator is because of the increase in optical loss due to the oblique and roughness of the side wall of the resonator. Consequently, the loss range of the broadband CPL also broadened for the same reason. Nevertheless, our experimental results accurately showed the trend of the simulation results. The almost perfect absorption of the spectral region of broadband CPL was broadened over that of a single CPL.

## 4 Conclusions

In conclusion, we demonstrated CPL through simulations and experimentally with respect to single and broadband resonators. In the simulation, both single and broadband CPLs were optimized at the PCN resonator. The designed single CPL demonstrated a perfect loss at 1550 nm with 1.76-nm FWHM. In addition, the broadband CPL showed a perfect loss region between wavelength of 1549 and 1551 nm and FWHM of 8.4 nm. We fabricated an optimized structure by using EBL on SOI. Furthermore, we measured an almost CPL-based perfect loss in both single and broadband PCN resonators. The maximum loss achieved using a single resonator was above 95 % at a wavelength of 1578 nm and FWHM of 5.4 nm. For broadband CPL, the loss region above 95 % ranges from 1577 to 1587 nm with an FWHM of 18.3 nm. The almost perfect absorption region by broadband CPL was observed to broaden more than that for the single resonator. The CPL using the PCN can be adopted in on-chip photonic integrated circuits for various applications such as sensors, modulators, and switches. Moreover, broadband CPL can overcome the limitations of a CPL caused by a narrow line width.

## References

[1] Baranov D. G., Krasnok A., Shegai T., Alù A., Chong Y. (2017). Coherent perfect absorbers: linear control of light with light. *Nat. Rev. Mater.*.

[2] Chong Y. D., Ge L., Cao H., Stone A. D. (2010). Coherent perfect absorbers: time-reversed lasers. *Phys. Rev. Lett.*.

[3] Wan W., Chong Y., Ge L., Noh H., Stone A. D., Cao H. (2011). Time-reversed lasing and interferometric control of absorption. *Science*.

[4] Rothenberg J. M. (2016). Experimental demonstration of coherent perfect absorption in a silicon photonic racetrack resonator. *Opt. Lett.*.

[5] Zhu W., Xiao F., Kang M., Premaratne M. (2016). Coherent perfect absorption in an all-dielectric metasurface. *Appl. Phys. Lett.*.

[6] Roger T. (2015). Coherent perfect absorption in deeply subwavelength films in the single-photon regime. *Nat. Commun.*.

[7] Zhang J. (2014). Coherent perfect absorption and transparency in a nanostructured graphene film. *Opt. Express*.

[8] Choi J., Noh H. (2018). Enhanced absorption by coherent control in a photonic crystal resonator coupled with a microfiber. *Opt. Lett.*.

[9] Wong Z. J. (2016). Lasing and anti-lasing in a single cavity. *Nat. Photonics*.

[10] Pichler K. (2019). Random anti-lasing through coherent perfect absorption in a disordered medium. *Nature*.

[11] Li S. (2014). An equivalent realization of coherent perfect absorption under single beam illumination. *Sci. Rep.*.

[12] Jin Y., Yu K. (2020). Broadband single-channel coherent perfect absorption with a perfect magnetic mirror. *Opt. Express*.

[13] Mock A. (2012). Low-power all-optical switch based on time-reversed microring laser. *IEEE Photonics J*..

[14] Li C., Qiu J., Ou J. Y., Liu Q. H., Zhu J. (2019). High-sensitivity refractive index sensors using coherent perfect absorption on graphene in the vis-NIR region. *ACS Appl. Nano Mater.*.

[15] Zhang Y., Wu F. P., Zhang H. F. (2022). Theoretical model of a RI THz sensor realized by coherent perfect absorption with optical phase modulation. *IEEE Sens. J.*.

[16] Meymand R. E., Soleymani A., Granpayeh N. (2020). All-optical AND, OR, and XOR logic gates based on coherent perfect absorption in graphene-based metasurface at terahertz region. *Opt. Commun.*.

[17] Slobodkin Y., Weinberg G., Hörner H., Pichler K., Rotter S., Katz O. (2022). Massively degenerate coherent perfect absorber for arbitrary wavefronts. *Science*.

[18] Wang C. (2022). Realization of broadband coherent perfect absorption of spoof surface plasmon polaritons. *Appl. Phys. Lett.*.

[19] Pu M. (2012). Ultrathin broadband nearly perfect absorber with symmetrical coherent illumination. *Opt. Express*.

[20] Baldacci L., Zanotto S., Biasiol G., Sorba L., Tredicucci A. (2015). Interferometric control of absorption in thin plasmonic metamaterials: general two port theory and broadband operation. *Opt. Express*.

[21] Noh H., Popoff S. M., Cao H. (2013). Broadband subwavelength focusing of light using a passive sink. *Opt. Express*.

[22] Li S. (2015). Broadband perfect absorption of ultrathin conductive films with coherent illumination: superabsorption of microwave radiation. *Phys. Rev. B*.

[23] Yoon J., Zhou M., Badsha M. A., Kim T. Y., Jun Y. C., Hwangbo C. K. (2015). Broadband epsilon-near-zero perfect absorption in the near-infrared. *Sci. Rep.*.

[24] Kim T. Y., Badsha M. A., Yoon J., Lee S. Y., Jun Y. C., Hwangbo C. K. (2016). General strategy for broadband coherent perfect absorption and multi-wavelength all-optical switching based on epsilon-near-zero multilayer films. *Sci. Rep.*.

[25] Bruno V. (2020). Dynamical control of broadband coherent absorption in ENZ films. *Micromachines*.

[26] Wang C., Sweeney W. R., Stone A. D., Yang L. (2021). Coherent perfect absorption at an exceptional point. *Science*.

[27] Guo T., Argyropoulos C. (2019). Tunable and broadband coherent perfect absorption by ultrathin black phosphorus metasurfaces. *J. Opt. Soc. Am. B*.

[28] Zhang Z. (2022). Dual-controlled tunable dual-band and ultra-broadband coherent perfect absorber in the THz range. *Opt. Express*.

[29] Luo P., Lan G., Nong J., Zhang X., Xu T., Wei W. (2022). Broadband coherent perfect absorption employing an inverse-designed metasurface via genetic algorithm. *Opt. Express*.

[30] Zhang H., Zhang H. (2022). Ultra-broadband coherent perfect absorption via elements with linear phase response. *Opt. Express*.

[31] Kotlicki O., Scheuer J. (2014). Wideband coherent perfect absorber based on white-light cavity. *Opt. Lett.*.

[32] Na J., Noh H. (2018). Investigation of a broadband coherent perfect absorber in a multi-layer structure by using the transfer matrix method. *J. Korean Phys. Soc.*.

[33] Braun M. M., Pilon L. (2006). Effective optical properties of non-absorbing nanoporous thin films. *Thin Solid Films*.

[34] Hutchinson N. J., Coquil T., Navid A., Pilon L. (2010). Effective optical properties of highly ordered mesoporous thin films. *Thin Solid Films*.

[35] Dong P., Liu C., Zhang L., Dai D., Shi Y. (2022). Reconfigurable add-drop filter based on an antisymmetric multimode photonic crystal nanobeam cavity in a silicon waveguide. *Opt. Express*.

[36] Zhang J., Pan B., Liu W., Dai D., Shi Y. (2022). Ultra-compact electro-optic modulator based on etchless lithium niobate photonic crystal nanobeam cavity. *Opt. Express*.

[37] Jalas D., Petrov A. Y., Eich M. (2014). Optical three-port circulators made with ring resonators. *Opt. Lett.*.

[38] Meng Z. M., Chen C. B., Qin F. (2020). Theoretical investigation of integratable photonic crystal nanobeam all-optical switching with ultrafast response and ultralow switching energy. *J. Phys. D Appl. Phys*..

[39] Duan B., Liu S., Liu X., Yu X., Wang C., Yang D. (2023). High-Q quasi-BIC in photonic crystal nanobeam for ultrahigh sensitivity refractive index sensing. *Results Phys.*.

[40] Liang F., Quan Q. (2015). Detecting single gold nanoparticles (1.8 nm) with ultrahigh- Q air-mode photonic crystal nanobeam cavities. *ACS Photonics*.

[41] Qiao Q., Xia J., Lee C., Zhou G. (2018). Applications of photonic crystal nanobeam cavities for sensing. *Micromachines*.

[42] Ozer Y., Kocaman S. (2018). Stability formulation for integrated opto-mechanic phase shifters. *Sci. Rep.*.

[43] Deotare P. B., McCutcheon M. W., Frank I. W., Khan M., Lončar M. (2009). Coupled photonic crystal nanobeam cavities. *Appl. Phys. Lett.*.

[44] Quan Q., Deotare P. B., Loncar M. (2010). Photonic crystal nanobeam cavity strongly coupled to the feeding waveguide. *Appl. Phys. Lett.*.

[45] McCutcheon M. W., Deotare P. B., Zhang Y., Lončar M. (2011). High-Q transverse-electric/transverse-magnetic photonic crystal nanobeam cavities. *Appl. Phys. Lett.*.

[46] Fr¨och J. E. (2020). Photonic nanobeam cavities with nanopockets for efficient integration of fluorescent nanoparticles. *Nano Lett*..

[47] Deng C. S., Peng H. G., Gao Y.-S., Zhong J. X. (2014). Ultrahigh-Q photonic crystal nanobeam cavities with H-shaped holes. *Phys. E*.

[48] Eichenfield M., Camacho R., Chan J., Vahala K. J., Painter O. (2009). A picogram- and nanometre-scale photonic-crystal optomechanical cavity. *Nature*.

[49] Yang S., Wu Y., Yang Y., Wang C., Tian H. (2021). High sensitivity and anti-external interference dual-parameter sensor based on a multimode slotted photonic crystal nanobeam cavity. *J. Mod. Opt.*.

[50] Zhao M. (2021). High Q chalcogenide photonic crystal nanobeam cavities. *IEEE Photonics Technol. Lett.*.

[51] Hu S., Weiss S. M. (2016). Design of photonic crystal cavities for extreme light concentration. *ACS Photonics*.

[52] Yang D. Q., Duan B., Liu X., Wang A.-Q., Li X. G., Ji Y. F. (2020). Photonic crystal nanobeam cavities for nanoscale optical sensing: a review. *Micromachines*.

[53] Liu L. (2022). Photonic crystal nanobeam cavity with a high experimental Q factor exceeding two million based on machine learning. *J. Lightwave Technol.*.

[54] Zhang Y. (2010). Photonic crystal nanobeam lasers. *Appl. Phys. Lett.*.

[55] Perahia R., Cohen J. D., Meenehan S., Alegre T. P. M., Painter O. (2010). Electrostatically tunable optomechanical ‘zipper’ cavity laser. *Appl. Phys. Lett.*.

[56] Lee J., Karnadi I., Kim J. T., Lee Y. H., Kim M. K. (2017). Printed nanolaser on silicon. *ACS Photonics*.

[57] Choi J., Noh H. (2021). Single-port coherent perfect loss in a photonic crystal nanobeam resonator. *Nanomaterials*.

[58] Wave Optics Module User's Guide (2022). ..

[59] Tatian B. (1984). Fitting refractive-index data with the Sellmeier dispersion formula. *Appl. Opt.*.

[60] Malitson I. H. (1965). Interspecimen comparison of the refractive index of fused silica*,†. *J. Opt. Soc. Am.*.

[61] Kim M. K., Kim J. Y., Kang J. H., Ahn B. H., Lee Y. H. (2011). On-demand photonic crystal resonators. *Laser Photonics Rev.*.

[62] Choi J., Noh H. (2021). Unidirectional incident wave for an electromagnetic wave simulation using the finite element method. *J. Korean Phys. Soc.*.

[63] Yvind K., Hvam J. M. (2010). High-efficiency, large-bandwidth silicon-on-insulator grating coupler based on a fully-etched photonic crystal structure. *Appl. Phys. Lett.*.

